# Stem-cell therapy for hearing loss: are we there yet?^☆^

**DOI:** 10.1016/j.bjorl.2019.04.006

**Published:** 2019-05-18

**Authors:** Luiz Gustavo Dufner-Almeida, Dayane Bernardino da Cruz, Regina Célia Mingroni Netto, Ana Carla Batissoco, Jeanne Oiticica, Rodrigo Salazar-Silva

**Affiliations:** a Universidade de São Paulo, Instituto de Biociências, Departamento de Genética e Biologia Evolutiva, São Paulo, SP, Brazil; b Universidade de São Paulo, Faculdade de Medicina, Laboratório de Otorrinolaringologia/LIM32, São Paulo, SP, Brazil

**Keywords:** Cellular therapy, Stem cells, Hair cells, Auditory neurons, Terapia celular, Células-tronco, Células ciliadas, Neurônios auditivos

## Abstract

**Introduction:**

Mammalian hair cells and auditory neurons do not show regenerative capacity. Hence, damage to these cell types is permanent and leads to hearing loss. However, there is no treatment that re-establishes auditory function. Regenerative therapies using stem cells represent a promising alternative.

**Objective:**

This article aims to review the current literature about the main types of stem cells with potential for application in cell therapy for sensorineural hearing loss, the most relevant experiments already performed in animals, as well as the advances that have been recently made in the field.

**Methods:**

Research included the databases PubMed/MEDLINE, Web of Science, Science Direct and SciELO, as well as gray literature. Search strategy included the following main terms: “stem cells”, “hair cells” and “auditory neurons”. Additionally, the main terms were combined with the following secondary terms: “mesenchymal”, “iPS”, “inner ear”, “auditory”. The research was conducted independently by three researchers.

**Results:**

Differentiation of stem cells into hair cells and auditory neurons has a high success rate, reaching up to 82% for the first and 100% for the latter. Remarkably, these differentiated cells are able to interact with hair cells and auditory neurons of cochlear explants through formation of new synapses. When transplanted into the cochlea of animals with hearing loss, auditory restoration has been documented to date only in deafferented animals.

**Conclusion:**

Advances have been more prominent in cases of auditory neuropathy, since partial improvement of auditory nerve conditions through cell-based therapy may increase the number of patients who can successfully receive cochlear implants.

## Resumo

**Introdução:**

Nos mamíferos, as células ciliadas e os neurônios auditivos não apresentam capacidade regenerativa. Assim, os danos a esses tipos celulares são permanentes e levam à perda auditiva. Contudo, como não há tratamento que restabeleça a função auditiva, as terapias regenerativas utilizando células-tronco representam uma alternativa promissora.

**Objetivo:**

Este artigo tem como objetivo revisar a literatura atual sobre os principais tipos de células-tronco com potencial para aplicação em terapia celular para perda auditiva sensorioneural, os experimentos mais relevantes já realizados em animais, bem como os avanços obtidos recentemente nessa área.

**Método:**

As pesquisas incluíram as bases de dados PubMed/MEDLINE, Web of Science, Science Direct e SciELO, além da literatura cinza. A estratégia de busca incluiu os seguintes termos principais: “stem cells”, “hair cells” e “auditory neurons”. Além disso, os termos principais foram combinados com os seguintes termos secundários: “mesenchymal”, “iPS”, “inner ear” e “auditory”. A pesquisa foi realizada de forma independente por três pesquisadores.

**Resultados:**

A diferenciação de células-tronco em células ciliadas e neurônios auditivos têm alta taxa de sucesso, chegando a 82% para o primeiro caso e 100% para o segundo. Notavelmente, essas células diferenciadas são capazes de interagir com células ciliadas e neurônios auditivos de explantes cocleares através da formação de novas sinapses. Quando transplantadas para a cóclea de animais com perda auditiva, a restauração da função auditiva foi observada, até o momento, apenas em animais com ablação do VIII nervo craniano.

**Conclusão:**

Os avanços têm sido mais proeminentes em casos de neuropatia auditiva. A melhora parcial das condições do nervo auditivo por meio de terapia baseada em células-tronco pode aumentar o número de pacientes candidatos a receber implantes cocleares com sucesso.

## Introduction

Hearing loss (HL) is a disorder with multiple causes that affects about 360 million people worldwide, according to the World Health Organization (WHO).^1^ As reviewed by Shearer et al.,^2^ HL may have environmental and/or genetic origin. Mammalian cochlear cells do not regenerate. Therefore, the loss of these cells, whether congenital or of late onset, due to genetic or environmental factors, causes irreversible hearing deficits.^3^ HL can be classified regarding many different criteria, but classification according to the region of the auditory system where the dysfunction is located is relevant to clinical management. Based on this criterion, there are three main types of HL: (i) conductive, when the abnormality is located in the external and/or middle ear region and results in a physical inability to conduct sound; (ii) sensorineural, when the affected region is in the inner ear and/or auditory neurons and/or central auditory pathways, and (iii) mixed, when HL results from the combination of conductive and sensorineural damage.

No clinical therapies currently exist to regenerate sensorineural cells loss or injury, but a variety of hearing assistive technologies can help individuals with hearing loss. Among these, there are personal sound amplification products (PSAPs) and cochlear implants (CIs). PSAPs are indicated for people with conductive deafness of any degree or sensorineural degree of mild to moderate degree. CIs are indicated for patients with severe to profound sensorineural deafness.^4^, ^5^ The PSAPs are different types of equipment that modify and amplify sound frequencies in which hearing is compromised. On the other hand, CIs are electronic prostheses partially implanted in the temporal bone and cochlea, capable of electrical stimulation of the ganglion cells of the auditory nerve.^6^

Knowledge about the auditory system development and about diseases affecting hearing is being constantly expanded and has enabled remarkable advances regarding gene and cellular therapies targeted to the hearing function. For the latter alternative, stem cells play a key role for restoring lost cochlear cells.

Stem cells are characterized by the properties of (i) self-renewal, *i.e.*, they are able to enter cell division indefinitely, and (ii) differentiation, *i.e.* are capable of modifying their phenotype and transforming into a variety of cell types, depending on their potential. This differentiation potential has been explored aiming for the regeneration of hair cells and auditory neurons. Three different types of stem cells are mainly employed for cell regeneration studies:(i) embryonic stem cells (ESCs), (ii) adult stem cells (ASCs) and (iii) induced pluripotent stem cells (iPSCs).^7^, ^8^, ^9^

ESCs are isolated from the inner cell mass of the mammalian blastocyst. These cells display unlimited proliferation capacity, *in vivo* and *in vitro*, and can differentiate into any tissue derived from the three primary germ layers, thus being classified as pluripotent cells. Although pluripotency poses an attractive feature for clinical application and cellular therapy, human ESCs are surrounded by drawbacks and ethical issues, such as the destruction of embryos for their isolation and the potential risk of immunological rejection when transplanted.^7^, ^10^

According to ISSCR (International Society for Stem Cell Research), ASCs or tissue-specific stem cells exist in completely formed organs or tissues. As they retain the capacity for self-renewal and differentiation into cells from the same tissue or organ in which they are located, they are classified as multipotent. Despite this limitation, adult stem cells are considered valuable because (i) they are easier to extract from tissue samples, (ii) face fewer ethical issues than human ESCs and (iii) are good candidates for use in cell therapy, since they can be obtained from adult patients in treatment, reducing risks of immunological rejection.^11^ A therapy based on the use of cells from the same individual would allow a significant advance in partial or total hearing regeneration in patients who do not present germline mutations causing deafness, since it would avoid treatments that require immunosuppression and eliminate complications resulting from rejection of tissues originated from other patients or embryos.

Cell reprogramming due to ectopic expression of specific transcription factors has so far been the classical way to obtain induced pluripotent stem cell lines (iPSCs) from any somatic tissue of almost any mammalian species. In general, pluripotency-related transcription factors, also known as “Yamanaka Factors” or OSKMs (OCT4, SOX2, KLF4 and MYC) can induce the formation of iPSCs. Among the infinity of applications for iPSCs, can be highlighted: cell therapy, modeling of polygenic and monogenic diseases, studies of allelic variation and of complex gene characteristics, as well as drug screening tests.^12^

**Figure 1:**
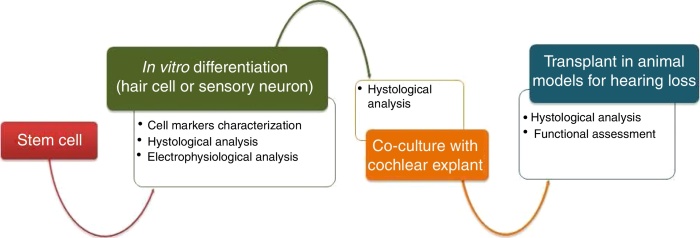
Schematics of main steps of cellular therapy for hair cell and auditory neuron regeneration.

Stem cells have the potential to contribute to the repair and restoration of the inner ear. For this strategy of cellular therapy to be a success, key steps are essential, such as the *in vitro* differentiation of different types of SCs into hair cells and/or auditory neurons, the functional integration of these differentiated cells in the cochlea and the evaluation of hearing restoration resulting from the therapy itself (Fig. 1). As a result, promising alternatives for hearing restoration have emerged, aiming to regenerate lost cochlear cells. Here we aim to review the main types of stem cells with potential for use in cell therapy for sensorineural HL, as well as the advances that have been recently made, focusing on the regeneration of hair cells and auditory neurons.

## Methodology

A structured review was performed from articles found in PubMed/MEDLINE, Web of Science, Science Direct and SciELO databases, as well as gray literature. Search strategy included the following main terms: “stem cell”, “hair cell”, “auditory neuron” and “sensory neuron”. Additionally, the main terms were combined with the following secondary terms: “mesenchymal”, “iPS”, “inner ear”, “auditory”. Only relevant articles published after 1995 were selected for this review. The research was conducted independently by three researchers.

## Results

### Obtaining hair cells and auditory neurons from stem cells

#### From embryonic stem cells (ESCs)

As described by Morsli et al.,^13^ cochlear development begins from the invagination of an ectodermal thickening in the otic placode, forming the otic vesicle. The multipotent cells present in the otic vesicle then differentiate into three different cell lines: (i) prosensorial line, which gives rise to the hair and support cells, (ii) proneural line, which originates the auditory and vestibular neurons and (iii) non-sensorial lineage responsible for the origin of cells with structural, secretory and/or absorption functions.^14^

The *in vitro* differentiation into hair or sensory neuronal cells from human embryonic stem cell is related to the expression of several well-characterized cell markers for each stage. However, obtaining auditory and hair neuronal cells presents a great challenge, both regarding the choice of culture media and of the specific factors required for direct differentiation of the otic progenitors to a desired cell type.

According to some studies, serum-free culture media containing N2 and B27 supplements is the starting point for ESC differentiation into Neuronal Progenitors (NPs), expressing characteristic markers of otic placode neuroblasts, such as Otx2, Brn3a, NeuroD, Pax2, Pax8 and nestin.^15^, ^16^, ^17^ Nevertheless, the differentiation efficiency of these supplements and cell culture substrates vary, even when the objective is to generate the same cell types. Shi et al.^16^ co-cultured human ESC with sensory epithelium of the mice cochlea in the presence of BMP4 factor. This co-culture was determinant for the expression of PERIPHERIN, GATA3, TRKB, TRKC and NGN1, markers of auditory neurons, in up to 20.6% of the cells. On the other hand, human immature hair-cell-like cells could also be obtained through FGF signaling and protracted culture with knockout serum replacement (KSR), although with low differentiation efficiency.^17^ In this case, only about 3% of hair cells expressed three or more hair cell markers, such as ATOH1, MYO7A, MYO15A and OTOF. Finally, it has been observed that human ESCs dissociated in a laminin layer and treated with FGF3 and FGF10 were able to generate two distinct types of otic progenitor colonies: otic epithelial progenitors (OEPs) and otic neural progenitors (ONPs). Submission of OEPs to “hair-cell” culture conditions produced hair-cell-like cells with 45% efficiency, expressing BRN3C and ATOH1 or MYO7A; in addition, submission of ONPs to “neuralizing” culture conditions produced sensory neurons with almost 100% efficiency, expressing BRN3A, β-tubulin III and NF200. It was also shown that these differentiated hair-cell-like cells and sensory neurons exhibit electrophysiological properties, demonstrating the viability of these cells to be responsive to mechanical stimuli and to transmit electrical stimuli.^15^

In order to obtain sensory neurons generated from ESCs, some authors focused on proving that such cells are able to project neural processes toward the cochlea, be it *in vitro* or *in vivo*. Shi et al.,^16^ for instance, showed that the sensory neural progenitors differentiated from human ESCs were able to mature into sensory neurons and sparsely innervate hair cells from murine organ of Corti explants, expressing β-tubulin and SYNAPSIN at the contact sites. Similar results were also observed by Nayagam et al.,^18^ who co-cultivated human neural progenitors with murine denervated cochlea explants for 10 days.

*In vivo* assays, Shi et al.^16^ transplanted neural progenitors, previously differentiated from human ESCs, into the base of the cochlea of deafferented gerbils. Sixty days after the procedure, the treated cochleae presented abundant new neuritis projecting toward the apex in the cochlear nerve trunk. Similarly, Chen et al.^15^ transplanted ONPs into the modiolus of deafferented gerbils and, three weeks after transplantation, observed ONPs expressing β-tubulin III and projecting neurites toward the organ of Corti. Ten weeks post-transplantation, these cells also migrated to the Rosenthal canal, made synaptic connections with hair cells and exhibited new projections toward the brainstem. However, the most important result of this study is that animals transplanted with ONPs were submitted to functional performance tests and exhibited hearing improvement of approximately 46%, with reduction of auditory thresholds of ouabain-treated animals from approximately 80 dB to 50 dB ten weeks after transplantation.

### From adult stem cells ASCs

#### Inner ear stem cells (IESCs)

The first evidence of the existence of SCs in the mammalian inner ear (IESCs) dates from 2003. Li et al.^19^ demonstrated the existence of cells from the posterior labyrinth or vestibule (peripheral vestibular system) with ability to form spheres, with properties of self-renewal and differentiation in cell types of the three germ layers. The researchers demonstrated that these ASCs, obtained specifically from the utricular macula, could be differentiated into ciliated cells, both *in vitro* and *in vivo*.

To date, there are no reports of the existence of IESCs in the cochlea of adult mammals. However, the existence of cells with SC properties could be evidenced from suspensions of cells dissociated from the organ of Corti of neonatal rats (P0).^20^ After cultivation, these cells expressed immature cell markers, similar to neuroepithelial IESCs. In addition, these cells were shown to form spherical colonies, named otospheres, and to be *in vitro* differentiated into both hair cells and support cells, albeit with an extremely low efficiency rate (about 1–2 cells per colony).

In a more detailed study with cell suspensions dissociated from the organ of Corti of human fetuses with 9–11 weeks of development,^21^ IESCs with expression of pluripotency markers were also identified. After culture establishment, several culture manipulation experiments were conducted to induce differentiation into neuronal cells and hair cells. The best protocols should ideally result in differentiated cells displaying not only cellular markers of these specific types, but also electrophysiological properties similar to these cell types *in vivo*. This was actually accomplished by means of trypsinization, with addition of Shh and bFGF for neuronal differentiation, with efficiency of 49%, and through supplementation of culture medium with EGF and retinoic acid for the hair cell differentiation, with efficiency of 56%.

As previously mentioned, the regenerative capacity of the mammalian cochlea is non-existent. However, if there is evidence of the existence of IESCs in the cochlea of both neonatal rats and human fetuses, as well as in the vestibule of adult mice, what is the reason behind the regenerative inability of the mammalian cochlea? In order to answer this question, Oshima et al.^22^ investigated the distribution of IESCs in the inner ear of mice with ages varying from P1 to P120. IESCs could be obtained from both the vestibular system represented by the utricular macula, saccular macula and ampullar crest, and the cochlear system represented by the organ of Corti, spiral ganglion and stria vascularis in mice up to P21, with a rapid decline in the population of these cells in the organ of Corti during the 2nd and 3rd week of life. When investigating P120 mice, IESCs were still identified in the three vestibular regions studied, but are apparently absent in the organ of Corti by this age. Two hypotheses have been raised for the depletion of this population of pluripotent cells: (i) actual loss of IESCs during the first 2 weeks of postnatal development,^23^ due to apoptosis in the great epithelial ridge (GER), an epithelium adjacent to the organ of Corti, which contains cells with proliferative capacity^24^; or (ii) loss of pluripotency markers by these cells during maturation of the cochlea, as a result from the differentiation process.^25^ In the latter case, otospheres formed from the organ of Corti would originate from post-mitotic supporting cells, since the ability of these cells to proliferate and *in vitro* differentiate into hair cells has been demonstrated. To date, none of the hypotheses was refuted.

Summing up, although the inner ear has stem cells, both in the vestibule and in the cochlea, after the development of the auditory system, this cell population is limited only to the vestibular region in adulthood. Added to this limitation are the great difficulty in obtaining these cells and the low efficiency of their differentiation into the desired cell types. Therefore, IESCs are currently more useful as an object of study of basic differentiation and ototoxicity screening, rather than an option for cellular therapy related to hearing disorders.

#### Mesenchymal stem cells (MSCs)

As reviewed by Keating,^26^ MSCs are defined as a phenomenon resulting from *in vitro* culture and were initially identified as a subpopulation of bone marrow cells. Criteria to define MSCs first included plastic adherence, *in vitro* differentiation into adipogenic, chondrogenic and osteogenic cells, expression of CD105, CD73 and CD90 markers and the absence of the hematopoietic markers.^27^, ^28^ The multi lineage differentiation capacity of mesenchymal stem cells makes them very valuable for cell-based medical therapies. Among many other useful properties of MSCs, they possess immunomodulatory properties which have made them attractive for regenerative medicine.^29^

Hair cell progenitors differentiated from mesenchymal stem cells were first obtained by Jeon et al.^30^ MSCs obtained from bone marrow of mice were initially transfected with EGFP-Math1 transcription factor and then differentiated into sensory precursors by culturing with EGF, IGF-1, bFGF, NT3 and BDNF for 14 days. At day 14, the authors observed that Math1 over-expression was able to induce expression of specific markers of auditory sensory epithelial cells, such as BRN3C, P27KIP, and JAGGED2. Expression of mature hair cell markers such as MYOSIN VIIA and ESPIN was also detected in 7.1% of the transfected cells, indicating differentiation of the murine MSCs into hair cells.

Hair cells could also be obtained, in culture, from bone marrow MSCs of rats. In the report of Qin et al.,^31^ bone marrow MSCs were first differentiated into neural progenitors through culture in serum-free medium containing EGF and bFGF. Then, after two weeks in medium with N2/B27, EGF and IGF-1, expression of Myosin VIIA could be detected through immunohistochemistry assays, suggesting that these cells are phenotypically similar to hair cells. Other transcription factors added to the culture medium of bone marrow MSCs also contributed to the production of neural progenitor cells, neurons and hair cells. Lee et al.,^32^ for instance, used BDNF, GDNF and NT3 factors in culture medium for 14 days. New hair cells were obtained, as confirmed by RT-qPCR and immunohistochemistry assays, showing that 3%–4% of the cells expressed MYOSIN VIIA.

In order to induce hair cell differentiation from human bone marrow MSCs, Duran Alonso et al.^33^ revealed some species-specific prerequisites for the process to be successful. Initially, they obtained neural progenitor cells with four different well-established protocols, expressing several neural progenitor markers such as NESTIN, SOX-2, OCT-4 and MUSASHI-1. Nonetheless, although combinations of EGF, bFGF and IGF-1 were already recognized as important to the induction of hair cell phenotype in rat bone marrow MSCs, the authors did not achieve the same results for the human counterpart. In this case, the induction of hair cell phenotype was only possible after culturing human cells in serum-free medium and with EGF and retinoic acid, a protocol previously used for the differentiation of human IESCs into hair cells.^21^ The differentiated human cells expressed specific markers of hair cells, such as ATOH1, MYOSIN VIA and CALRETININ, with maximum efficiency of 17.5%.^33^

Hair cells could also be differentiated from MSCs obtained from other tissues than bone marrow, such as adipose tissue. Lin et al.,^34^ for instance, reprogrammed murine adipose-derived MSCs into hair cell progenitors by using a combination of protein transfection, adenovirus and co-culture with neurons. MSCs were transfected with adenovirus expressing ATOH, a key transcription factor for hair cell development, fused to EGFP (enhanced green fluorescent protein). After two days, all EGFP-tagged cells expressed the hair cell marker MYOSIN VIIA. Approximately 82% of the cells submitted to Atoh1 over-expression had protrusions on their cell surface, similar to stereocilia. It is important to highlight that Lin's group was the first to obtain hair cells directly from multipotent stem cells without prior differentiation into otic progenitor cells, showing that the differentiation process into hair cells can be accelerated.

Regarding obtention of auditory neurons from MSCs, Duran Alonso et al.^33^ first differentiated human bone marrow MSCs into neural progenitors and then induced sensory neuron phenotype through supplementation of the medium culture with SHH, retinoic acid, BDNF, NT-3 and bFGF. By the end of the differentiation protocol, the authors observed expression of GATA-3, SOX2, NGN-1 and ISLET-1 sensory neuron markers in up to 47% of the cells. In the study of Lee et al.,^32^ the differentiation process consisted in incubating rat bone marrow MSCs for 5 days in culture medium supplemented with DMEM/F12, N2, B27, EGF and IGF-1, for neuronal progenitor phenotype induction. The neuronal progenitors were then differentiated into auditory neurons by replacing the culture medium with basic neurotrophic factor medium containing GDNF, BDNF and NT-3. In the end of the experiment, RT-qPCR and immunofluorescence assays confirmed the expression of neuronal markers NeuN and β-tubulin III in 7%–8% of the differentiated cells.

#### From human induced pluripotent stem cells (iPSCs)

Aiming to develop a cellular model for the study of Varicella Zoster Virus (VZF) pathogenesis, Lee et al.^35^ focused on the generation of sensory neurons *in vitro* from iPSCs, since VZF keeps itself latent in sensory ganglia. For this purpose, iPSC cells differentiated from fibroblasts were exposed to small inhibitory molecules and retinoic acid for 10 days, converting the pluripotent cells into neural progenitor cells, with expression of neural progenitor markers PAX6 and NESTIN. Following culture for 2 weeks in culture medium rich in growth factors, 80% of the cells expressed β-tubulin III, a neuron marker, while 15% of the cells co-expressed BRN3A and PERIPHERIN, sensory neuron markers. The iPSC-differentiated sensory neurons were also functional, being able to generate action potentials in response to depolarization.

Gunewardene et al.^36^ were also successful at establishing *in vitro* neurosensory cell lines from two human iPSC lines derived from foreskin. The cells were subjected to the following stepwise differentiation process:(i) neuronal induction, using Noggin with bFGF; (ii) neurosphere induction, using EGF and bFGF; (iii)neural crest induction, treated with a Rho kinase inhibitor; and, finally, (iv) induction of sensory neurons by culturing only with NBM. Neurosensorial cells derived from human iPSC expressed markers of dorsal rhombencephalon (PAX7), otic placode (PAX2), proneurosensory domain (SOX2), neural ganglia (NEUROD1, BRN3A, ISLET1, β-tubulin III, NFM), and sensory neurons (GATA3 and VGLUT1) over the time period examined, with the highest levels of expression observed on the 35th day of *in vitro* differentiation. The differentiated sensory neurons were also electrophysiologically active, although the activity pattern seen was more similar to auditory neurons of early postnatal mice. However, it is worth noting that, as reported in studies with human iPSC, the levels of mRNA expression observed were always variable among different human iPSC strains, among samples from the same lines and also when iPSCs were compared to human ESC controls. This variation, as suggested by the authors, may be closely related to genetic and epigenetic mutations resulting from the random integration of lentivirus into the genome during pluripotency induction process.

After standardization of the neuronal differentiation process as described above, the authors proceeded in the evaluation of the viability of the differentiated sensory neurons from two different lines of iPSCs in innervating hair cells ex vivo.^37^ After 10 days of co-culture, the neural progenitors were able to emit projections toward hair cells of the cochlear explant with up to 94.2% efficiency but in a disordered manner, a pattern of branching similar to that seen in auditory neurons at the beginning of development. Immunofluorescence assays also revealed synapses between the iPSC-derived neurons and hair cells, as indicated by the expression of SYNAPSIN-1. Finally, the authors observed that less differentiated neurons (21 days of *in vitro* differentiation) presented more synapses with a greater number of hair cells when compared to more differentiated neurons (28 days of *in vitro* differentiation), showing the importance of the differentiation phase of the neural progenitor chosen for transplantation to the maximization of the functional integration of these cells into the cochlear tissue.

Regarding iPSCs differentiation into hair cells, an elegant work was conducted by Oshima et al.^38^ iPSCs derived from fibroblasts of Atoh1/nGFP transgenic mice were cultured for 5 days with a Wnt pathway inhibitor, a Smad3 inhibitor, and IGF-1, followed by exposure to bFGF for 3 days. Otical progenitors were obtained and then differentiated into hair cells through co-culture with mitotically inactivated chicken utricle stromal cells. The overall hair cell differentiation efficiency, however, was low: about 12% of the iPSC derived hair cells expressed of MYO VIIA, while only 2.6% expressed both MYO VIIA and ESPIN. These iPSC-derived hair cells were both morphologically and electrophysiologically similar to immature hair cells. Finally, co-culture experiments revealed that sensory neural ganglia harvested from neonatal mice emit neural projections towards the IPSC-derived hair cells, with synapses confirmed by SYNAPTOPHYSIN (SYN) expression. On the other hand, Chen et al.^39^ were more successful in obtaining human iPSC-derived hair cells. These authors submitted iPSCs derived from human urinary cells to the same hair cell differentiation protocol used for ESCs,^15^ obtaining OEPs which, after employment of “hair-cell” culture conditions, produced hair-cell-like cells up to 50% efficiency. *In vitro* experiments showed that murine spiral ganglion neurons are able to project neurites toward the human iPSC-derived hair-cell-like cells and form synapses. Finally, for the first time in the literature, these OEPs derived from human iPSCs were transplanted into Slc26a4-null mice, animals that suffer hearing loss due to loss of function of pendrin that impairs hair cell functionality. Four weeks post-transplantation, new hair cells were identified in the organ of Corti, although in extremely small numbers. Despite not being able to arrange regularly as native hair cells, the transplanted cells expressed MYO VIIA and were targeted by native spiral ganglion neurons for synapses. Unfortunately, the transplanted animals were not submitted to functional evaluation to check if there was any hearing improvement.

### Pitfalls and ongoing questions about cellular therapy in hearing loss

There is no doubt about the enormous potential of cell therapy in the treatment of diverse pathologies that require regeneration of damaged cells and tissues, such as neurodegenerative diseases,^40^ muscular dystrophies^41^ and deafness. In addition to the most obvious obstacles to be overcome, which include the possibility of tumor formation and the rejection of transplanted cells by the recipient's immune system, other important aspects about the success of a therapy directed to deafness should also be taken in consideration.

The first point is the efficiency of the differentiation of SCs transplanted into hair cells or auditory neurons. Despite the constant expansion of the knowledge about molecular biology and embryonic development of the inner ear and its specific cell types, a very limited efficiency of differentiation is generally observed for the desired cell types. However, this adversity has been addressed, following the example of Liu, et al.,^42^ which, based on gene expression profiling studies during all differentiation stages of hair cells, were able to identify sets of molecular markers specific for IESCs, progenitor cells and hair cells. This should significantly increase the efficiency in identifying and differentiating SCs into mature hair cells.

The second issue is the survival of the transplanted cells after their insertion into the patients’ cochlea, since this would influence the number of transplantations necessary to obtain and to maintain the desired final auditory recovery. To date, transplantations have only been made in animal models for the regeneration of auditory neurons, and the highest survival rate was 94% of transplanted cells, 10 weeks after transplantation.^21^

A third point that should be considered is the functional integration of SCs transplanted to the patient's auditory system. For the time being, among all the transplantation studies for regeneration of auditory neurons, only one presented quantitative data regarding this issue, in which in the group of 50% of transplanted cells that survived one week after transplantation, only 2.3% expressed a neural glutamatergic marker of auditory neurons.^43^

Finally, there are other questions brilliantly raised by some authors^44^, ^45^ which deserve future investigation, such as (i) how the transplanted cells will distribute along the cochlear duct and regenerate the organ of Corti, (ii) the possibility that endolymph, the liquid that fills the compartment that houses the organ of Corti, can be toxic to the transplanted cells due to its high content of potassium, (iii) the fact that, for a successful integration of the transplanted cells, there is a need for a breakdown in cell adhesion between the remaining hair and support cells in the organ of Corti; finally, (iv) in the case of hair cell regeneration, there is a possibility for SCs to integrate ectopically into the cochlea and impair the cochlear function. It is necessary for the transplanted cells to integrate and to position themselves correctly on the basilar membrane, so that they can be correctly activated, a quite unlikely event considering the high complexity of the organ of Corti cytoarchitecture.

## Conclusions

Significant advances have been made on studies about cellular differentiation, both of hair cells and of auditory neurons (Fig. 1). However, the success rates of the auditory neurons (Table 1) regeneration protocols are generally higher than in hair cells (Table 2), with some studies already focusing on the transplantation of these cells into the cochleae of animal models, and obtaining very encouraging results. It has also been seen that restoration of hearing through regeneration of hair cells, although equally important, is still far from being achieved. Few studies have performed transplantations of differentiated hair cells into cochleae of animal models and, therefore, there are not many reports of the effective ability to restore hearing using these cells. In addition, the regeneration of hair cells presents some obstacles difficult to circumvent, such as the high complexity of the organ of Corti cytoarchitecture, which hinders the correct integration of the transplanted cells into the sensory epithelium. An alternative that has been very recently explored is the transdifferentiation of support cells into hair cells, a process first observed in the auditory system of birds and that, in theory, would overcome the mentioned problems.^46^, ^47^, ^48^, ^49^

**Table 1:** Summary of the advances in the main steps for auditory neuron regeneration.

	Auditory neuron			
	*In vitro* differentiation from			
	ESC^15^, ^16^	IESC^21^	MSC^32^, ^33^	IPSC^35^, ^36^
Cellular markers expressed	PERIPHERIN, GATA3, TRKB, TRKC and NGN	NEUROG1, POU4F1 β-TUBULIN III and PVALB	GATA-3, NGN-1, NeuN, ISLET-1 and B-TUBULIN III	β-TUBULIN III, BRN3A and PERIPHERIN
Efficiency	20.6%–100%	49%	7%–47%	80%–100%
Electrophysiological tests?	Performed	Performed	Not performed	Performed
	Co-culture with cochlear explants			
	ESC^16^, ^18^	IESC	MSC	IPSC^37^
Cellular markers expressed	β-TUBULIN and SYNAPSIN	–	–	SYNAPSIN-1, PAX7, PAX2, SOX2, NEUROD1, BRN3A, ISLET1, β-TUBULIN III, NFM, GATA3 and VGLUT1
New neuritis projections between stem cells and cochlear cells?	Yes	–	–	Yes, improvement of 94.2%
	Transplantation in animal models for hearing loss			
	ESC^15^, ^16^	IESC	MSC	IPSC
Survival rate	22.87%–94.9%	–	–	–
Post-transplant analysis time range	21–70 d	–	–	–
Hearing recovery?	Yes, partial	–	–	–

**Table 2:** Summary of the advances in the main steps for hair cell regeneration.

	Hair cell			
	*In vitro* differentiation from			
	ESC^15^, ^16^, ^17^	IESC^19^, ^21^	MSC^30^, ^33^, ^34^, ^35^	IPSC^38^, ^39^
Cellular markers expressed	ATOH1, MYO7A, MYO15A, OTOF, BRN3A, β-TUBULIN III and NF200	ATOH1, MYOSIN VIA, ESPIN and CALRETININ	CD105, CD73, CD90 and MYOSIN VIIA	MYO VIIA and ESPIN
Efficiency	3.3%–45%	10.7%–56%	3%–82%	2.6%–50%
Electrophysiological tests?	Conducted	Conducted	Not conducted	Conducted
	Co-culture with sensory neural ganglia			
	ESC	IESC	MSC^33^	IPSC^38^, ^39^
Cellular markers expressed	–	–	–	SYP
New neurites projections between stem cells and cochlear cells?	–	–	–	Yes
	Transplant in animal models for hearing loss			
	ESC	IESC	MSC	IPSC^38^, ^39^
Survival rate	–	–	–	Not assessed
Post-transplant analysis time range	–	–	–	28d
Hearing recovery?	–	–	–	Not assessed

Therefore, given the great improvement in the attempts of regeneration of auditory neurons, we believe that more studies should be carried out in this path, focusing on the efficiency of the functional integration of transplanted cells in the auditory system of animal models. Thus, it may be that we are closer to treating the cases of auditory neuropathy, that is, cases of hearing impairment characterized by the normal functioning of hair cells and abnormal or absent functioning of auditory neurons,^49^ which make up 15% of hearing loss cases.^50^ Such procedures could at least enable more individuals with hearing loss to receive CI, since one of the prerequisites for this type of treatment is the presence of healthy auditory neurons in the patient.

## Funding

Fundação de Amparo à Pesquisa do Estado de São Paulo (FAPESP) – CEPID – Centro de Pesquisa sobre o Genoma Humano e Células-Tronco (2009/09473-3 and 13/08028-1). Conselho Nacional de Desenvolvimento Científico e Tecnológico (CNPq, grant 133182/2015-0, Coordenação de Aperfeiçoamento de Pessoal de Nível Superior (Capes, grants 33002010070P8 and PROEX (001)).

## Conflicts of interest

The authors declare no conflicts of interest.
